# Are All Primary Immunodeficiency Disorders Inborn Errors of Immunity?

**DOI:** 10.3389/fimmu.2021.706796

**Published:** 2021-07-21

**Authors:** Rohan Ameratunga, Hilary Longhurst, Klaus Lehnert, Richard Steele, Emily S. J. Edwards, See-Tarn Woon

**Affiliations:** ^1^ Department of Clinical Immunology, Auckland Hospital, Auckland, New Zealand; ^2^ Department of Virology and Immunology, Auckland Hospital, Auckland, New Zealand; ^3^ Department of Molecular Medicine and Pathology, School of Medicine, Faculty of Medical and Health Sciences, University of Auckland, Auckland, New Zealand; ^4^ Department of Medicine, School of Medicine, Faculty of Medical and Health Sciences, University of Auckland, Auckland, New Zealand; ^5^ School of Biological Sciences, University of Auckland, Auckland, New Zealand; ^6^ B Cell Differentiation Laboratory, Department of Immunology and Pathology, Central Clinical School, Monash University, Melbourne, VIC, Australia

**Keywords:** inborn error of immunity, primary immunodeficiency, THI, common variable immunodeficiency disorders, THA

## Introduction

It is almost 70 years since the first description of Bruton’s agammaglobulinemia (1). In the last decade there has been a rapid increase in the rate of discovery of new genetic defects in primary immunodeficiency disorders (PIDs), largely due to the advent of Next Generation Sequencing (NGS) (2, 3). NGS utilises massively parallel sequencing to analyse either the exome (WES) or the entire genome (WGS). The International Union of Immunological Societies (IUIS) expert committee of the WHO has curated over 400 such disorders (2).

This sequencing revolution has had profound benefits (and some disadvantages) for patients and their clinicians as well as scientists seeking to identify new disorders (4, 5). As termed by Robert Good, these “experiments of nature”, have offered unique scientific insights into functioning of the immune system (6).

The many overlapping benefits of genetic confirmation for patients include certainty of diagnosis, prognostic insights and specific treatments (5). It has ushered in the era of personalised medicine. Identification of a gain for function mutation of *PIK3CD* for example, may lead to specific treatments such as idelalisib in addition to subcutaneous or intravenous immunoglobulin (SCIG/IVIG) replacement.

With the rapid increase in the discovery of new genetic defects, there has been a move to name these conditions inborn errors of immunity (IEI) (2). The Merriam Webster dictionary states Inborn is “being a part of the innermost nature of a person or thing” and synonyms include *congenital*, *hereditary* and *inherited*. Inborn thus implies these conditions are genetic and inherited, which will be transmitted to future generations. Errors in this context infer mutations, which are pathogenic and underlie the phenotype of the patient.

**Figure 1:**
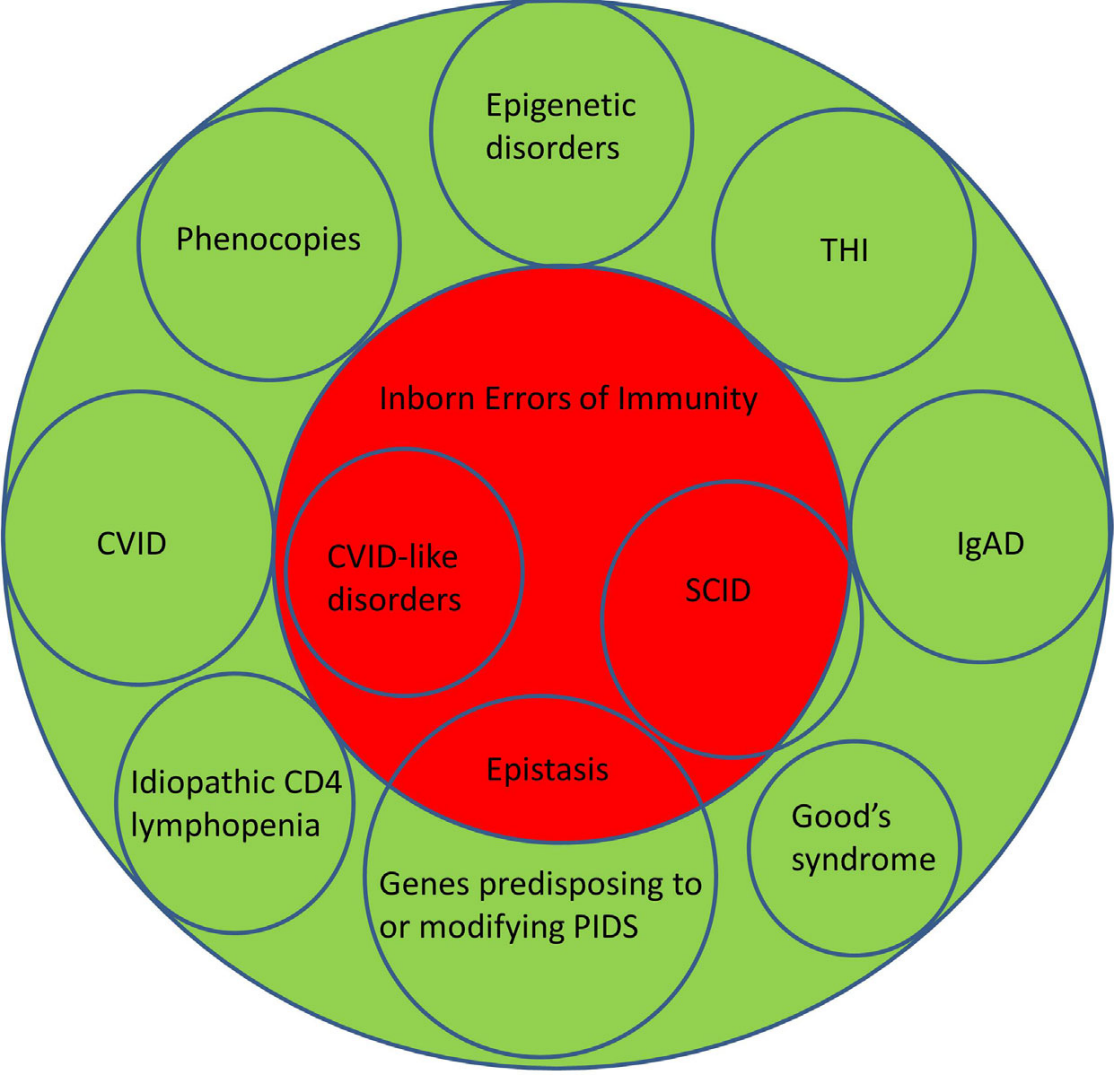
Illustrating the relationship between Primary Immunodeficiency Disorders (green) and Inborn Errors of Immunity (red). CVID, Common Variable Immunodeficiency Disorders; IgAD, IgA deficiency; SCID, Severe Combined Immunodeficiency; THI, Transient Hypogammaglobulinemia of Infancy.

Although there is an argument for changing the name from PIDs to IEI, or using these terms interchangeably, there are several caveats. Three of the most common conditions, which numerically comprise the majority of patients with PIDs, do not currently have a definable genetic basis; IgA deficiency (IgAD), Common Variable Immunodeficiency Disorders (CVID) and Transient Hypogammaglobulinemia of Infancy (THI). Other well-recognised conditions, which do not have a genetic explanation at this time, include Good’s syndrome and CD4 lymphopenia. Even within well-defined phenotypes such as agammaglobulinemia with absent B cells or Severe Combined Immunodeficiency (SCID), not all patients have a genetic explanation for their disorder (**Figure 1**). This is a perspective on why these terms are not currently interchangeable and why it may be premature to abandon the term PID in favour of IEI (**Table 1**).

**Table 1:** PIDs for which a causative genetic basis has not been identified in all patients.

Disorder	Genetic basis	Comment
IgA deficiency	Unknown	Commonest PID (7)
CVID	Unknown (by definition)	Patients with causative genetic defects are reclassified as CVID-like disorders (3, 8)
THI	Unknown	Retrospective diagnosis (9)
Idiopathic CD4 lymphopenia	Unknown	Increasingly recognised with the advent of new-born screening (10)
Good’s syndrome	Unknown	Thymic abnormalities may contribute to the combined immunodeficiency (11)
SCID (and many other similarly well- characterised disorders)	Mostly known	Not all patients with a SCID phenotype have an identifiable mutation
Phenocopies	Acquired disorders	Can be identified by genetic sequencing (2)
Epigenetic defects	Acquired disorders	Difficult to identify (12)

CVID, Common Variable Immunodeficiency Disorders; SCID, Severe Combined Immunodeficiency; THI, Transient Hypogammaglobulinemia of Infancy.

## IgA Deficiency

IgA deficiency (IgAD) is the most common PID in humans. IgAD can occur in the context of other well-defined PIDs such as SCID or X-linked hyper IgM syndrome or as a sporadic disorder. The frequency of sporadic IgAD may be as high as 1:358 in blood donors (7). The majority of patients with sporadic IgAD are asymptomatic, presumably because other arms of the immune system can compensate for the lack of IgA in serum and on mucosal surfaces. The genetic basis for sporadic IgAD is not understood (13). While there is an increased proportion of IgAD patients with *TNFRSF13B*/TACI mutations, the prevalence of such variants exceed that of sporadic IgAD (14). Application of the American College of Medical Genetics (ACMG) criteria indicates TACI mutations are not the cause of sporadic IgAD (15).

## Common Variable Immunodeficiency Disorders

CVIDs are the most common symptomatic PID in adults and children. The prevalence of CVID varies between 1:25 000 to less than 1:100 000 in the general population. Although there may be ascertainment bias, these disorders are less common in Asian and African populations. Reports from developed Asian countries with advanced healthcare systems, such as Taiwan, Japan or South Korea indicate a very low frequency of CVID (16–19). In contrast, a recent study has shown an unexpected high prevalence of CVID in the indigenous Māori of New Zealand (20). The basis for these ethnic-specific rates is not known.

By definition, the genetic causes for CVID are unknown (21–23). There are now over forty genetic variants which are associated with CVID (3, 8, 24). Some of these are causative (*NFKB1, NFKB2* etc) while others predispose to or modify disease severity (*TNFRSF13B*/TACI, *TNFRSF13C*/BAFFR, *MSH5* etc.) in CVID. Sometimes mutations in genes such as *RAG* can lead to atypical presentations which might be considered CVID-mimics. If patients have a causative genetic defect, they are now deemed to have a CVID-like disorder with their own specific mutation. Patients with CVID-like disorders could be considered to have an IEI (**Figure 1**).

In consanguineous societies, the proportion of patients with CVID-like disorders is approximately 60-70% (25, 26). Patients from such countries have highly penetrant autosomal recessive monogenic disorders often presenting in childhood. In contrast, only 25% patients from non-consanguineous populations have an underlying causative mutation, mostly late-onset autosomal dominant disorders (27). In non-consanguineous societies, this leaves 75% of CVID patients without a genetic explanation and IEI would seem an incorrect term for these patients.

CVID-like disorders are characterised by marked genetic, allelic and phenotypic heterogeneity (8). It is apparent that several causative genetic defects lead to the same phenotype of recurrent infections and autoimmune disorders (locus heterogeneity, genocopy). Similarly, there can be marked phenotypic variation in the same family carrying the identical mutation. In one of the three families where mutations of *NFKB1* were first described, one brother was completely asymptomatic, while his sister passed away from late onset combined immunodeficiency (LOCID). Other members of the family had predominantly autoimmune disorders (28, 29). This phenotypic heterogeneity of CVID-like disorders is compatible with IEI as the majority of patients with causative genetic defects would be expected to become symptomatic at some stage of their lives. It is likely environmental triggers such as Herpes simplex infections may alter their prognostic trajectory.

To complicate the genetics of CVID and CVID-like disorders, a group of genes predispose to or modify disease severity in CVID and CVID-like disorders (30). These genes include *TNFRSF13B*/TACI, *TNFRSF13C*/BAFFR, *MSH5* etc. While there is a higher prevalence of mutations of these genes in patients with CVID and CVID-like disorders, their frequency in the general population far exceeds that of CVID (31). The majority of healthy individuals carrying these mutations will not suffer CVID. While these could be considered variants and are inherited, they are not causative and should not be considered IEI. The ACMG criteria cannot be applied to genes which predispose to or modify CVID (15).

This was recently illustrated in a family presenting with a severe CVID-like disorder (32). The proband had mutations of both *TNFRSF13B*/TACI as well as *TCF3*. Family studies showed it was the *TCF3* mutation, which segregated with the CVID-like disorder, while the *TNFRSF13B*/TACI mutation modified the disorder in an epistatic fashion (33). Epistasis is the synergistic interaction of two or more genes which can modify disease severity or lead an entirely different phenotype (34). This family illustrates the complexity of CVID and CVID-like disorders and the need for accurate description of the underlying genetics. Individuals from this kindred carrying the *TCF3* mutation could be considered to have an IEI but not those with *TNFRSF13B*/TACI, which modified the severity of the disorder.

## Transient Hypogammaglobulinemia of Infancy

Transient hypogammaglobulinemia of infancy is an important cause of reduced immunoglobulin levels in early life (35). Current thinking is that there is delayed maturation of IgG production in these infants. By definition, the IgG normalises over time and THI is a retrospective diagnosis. Recently it has been shown that the majority of patients do not normalise their IgG until after four years of age (9).

The genetic basis of THI is currently not understood. This is likely to be a common PID but may not be recognised as many patients are asymptomatic. Again, it is inaccurate to term these infants as having an IEI.

## Other Idiopathic Disorders With a Probable Genetic Basis

There are many other well-defined disorders where the genetic basis has not been identified. This includes patients with Good’s syndrome, a combination of thymoma, CD4 lymphopenia and hypogammaglobulinemia (11). Most patients with Good’s syndrome are diagnosed following a CT scan of the thorax, when the thymoma is identified.

Following the advent of new-born screening it has become apparent many patients with low numbers of T cell receptor excision circles (TRECs) have idiopathic T cell lymphopenia rather than Severe Combined Immunodeficiency (SCID) (10). The long-term prognosis of these infants is not known. The genetic basis for persistent idiopathic T cell lymphopenia is not understood and it would not be accurate to label this condition as an IEI (36).

## Phenocopies

The WHO/IUIS committee has recognised a group of disorders called phenocopies, which do not easily fit with IEI (2). Phenocopies are caused by discrete clones of cells, which proliferate and reproduce the phenotype of the disorder. There are many examples of these somatic disorders including the autoimmune lymphoproliferative syndrome (ALPs). While these conditions have a genetic basis, they cannot be inherited and do not meet the inborn component of IEI. It may still be appropriate to call them PIDs as there is no secondary cause.

## Epigenetic Disorders

Monozygotic twins discordant for CVID have been described (12). The authors speculated the explanation was epigenetic changes in several genes including *TCF3*. It will be important to show these methylation patterns are stable over time and segregate with the phenotype of the affected twin. It seems likely other PIDs will be identified in the future, where epigenetic changes are the basis of the disorder. Epigenetic changes are not considered IEIs but would be considered PIDs.

## Discussion

This essay has examined the interchangeability of the terms PID and IEI. There are many reasons why the term primary immunodeficiency disorder is currently preferred to inborn error of immunity. This is not merely a matter of semantics, as it has important implications for scientific accuracy as well as clinical management of these patients.

Unlike IEI, PID does not necessarily imply there is an inherited genetic basis for the disorder and could include phenocopies. The aberrant clone of cells harbouring the mutation is directly responsible for the clinical manifestations of disease. In secondary causes such as lymphoma, the immune defect is consequent to the malignant process. Phenocopies can thus be considered PIDs as they are the cause of the disorder, but not IEIs as they cannot be inherited despite having a genetic basis.

Epigenetic disorders cannot be easily identified by current sequencing technologies but may be more prevalent than is currently perceived. If more disorders are shown to have an epigenetic basis, the term IEI will be inappropriate as these conditions cannot be inherited. They will however remain PIDs as there is no secondary cause for the disorder.

Scientific integrity is important. Given that the genetic basis of CVID is by definition not known, stating patients with CVID have an IEI is inaccurate. In addition to scientific inaccuracy, semantics can also adversely affect patient care. As seen in the description of CVID and CVID-like disorders, the genetics of PIDs are complex (3, 24, 37). It is very important for patients to be appropriately counselled, particularly in non-consanguineous populations that there is a greater chance a causative mutation will not be identified. Such genetic counselling is an essential part of pre-analytical testing. If patients with CVID are advised they have an IEI, it may create unrealistic expectations that a causative genetic defect will be identified.

Furthermore, it may create anxiety as IEI more than PID might indicate the disorder will be passed to the next generation. PIDs, like many familial conditions are associated with parental guilt. This may be exacerbated if the genetic basis is not identified after testing. PIDs in contrast do not imply all patients have a genetic basis for their disorder, as there are other pathogenic mechanisms. Classifying CVID as a subset of PIDs is both scientifically accurate and is more helpful in managing patient expectations during pre-analytical counselling (**Figure 1**).

IEI also implies greater scientific understanding than is currently the case for many disorders. Asymptomatic IgAD likely comprises the majority of patients with PIDs. As noted above, the genetic basis for sporadic IgAD is unknown. As with CVID, it is scientifically more accurate to term these conditions PIDs than IEIs. Numerically, symptomatic and asymptomatic patients with sporadic IgAD far exceed all other PIDs and cannot be termed IEIs.

Similarly, although rare familial cases of THI have been described in siblings, the vast majority are sporadic (9). The genetic basis for familial THI is unknown. The use of the term IEI could cause unnecessary parental anxiety and guilt if the index child has suffered invasive infections and has needed SCIG/IVIG. In this context IEI implies a greater understanding of THI than is the case.

One argument for using IEI is the case of gain of function (GOF) mutations leading to autosomal dominant disorders. Such heterozygous cases lead to a phenotype, which is often very different from patients with loss of function mutations in the same gene. Although at first glance, the term PID may not seem to fit well, these conditions could be considered to be an immunodeficiency of regulatory elements of gene function. A similar argument could be made for patients with CVID-like disorders presenting with autoimmunity with a minimal history of infections.

Inspite of these differences, there are however areas of agreement between the terms PID and IEI. Both exclude secondary causes such as infections, gut disease, malignancy, renal immunoglobulin loss etc. (38). Generally secondary causes such as malignancy can be easily distinguished from PIDs. The age of the patient is helpful in considering the likely secondary causes. The passage of time and family history can usually help distinguish secondary causes from a primary disorder.

In the last decade, there has been rapid progress in the identification of the genetic basis of many PIDs. If there is another future genetic revolution akin to NGS, it is possible all patients with PIDs will have a genetic diagnosis. Apart from patients with phenocopies and epigenetic changes, the two terms PID and IEI may be interchangeable.

All current definitions of CVID exclude a known cause for hypogammaglobulinemia (21–23). Counterintuitively, if the genetic basis of CVID was understood in all patients, the disorder will cease to exist (8). All such patients with CVID-like disorders will have their own genetic defect/IEI. It would seem reasonable to revisit this topic periodically, particularly if there have been major advances in technology.

## Author Contributions

RA wrote the first draft. All authors contributed to the article and approved the submitted version.

## Conflict of Interest

The authors declare that the research was conducted in the absence of any commercial or financial relationships that could be construed as a potential conflict of interest.
